# Transcriptomic changes during TGF-β-mediated differentiation of airway fibroblasts to myofibroblasts

**DOI:** 10.1038/s41598-019-56955-1

**Published:** 2019-12-30

**Authors:** Erin Joanne Walker, Deborah Heydet, Timothy Veldre, Reena Ghildyal

**Affiliations:** 1 Centre for Research in Therapeutic Solutions, Faculty of Science and Technology, University of Canberra, Bruce, ACT 2617 Australia; 2 Present Address: Department of Respiratory and Sleep Medicine, The Canberra Hospital, Garran, ACT 2605 Australia

**Keywords:** Cellular signalling networks, Experimental models of disease

## Abstract

Asthma is the most common chronic lung disease in children and young adults worldwide. Airway remodelling (including increased fibroblasts and myofibroblasts in airway walls due to chronic inflammation) differentiates asthmatic from non-asthmatic airways. The increase in airway fibroblasts and myofibroblasts occurs *via* epithelial to mesenchymal transition (EMT) where epithelial cells lose their tight junctions and are transdifferentiated to mesenchymal cells, with further increases in myofibroblasts occurring via fibroblast-myofibroblast transition (FMT). Transforming growth factor (TGF)-β is the central EMT- and FMT-inducing cytokine. In this study, we have used next generation sequencing to delineate the changes in the transcriptome induced by TGF-β treatment of WI-38 airway fibroblasts in both the short term and after differentiation into myofibroblasts, to gain an understanding of the contribution of TGF-β induced transdifferentiation to the asthmatic phenotype. The data obtained from RNAseq analysis was confirmed by quantitative PCR (qPCR) and protein expression investigated by western blotting. As expected, we found that genes coding for intermediates in the TGF-β signalling pathways (*SMAD*s) were differentially expressed after TGF-β treatment, *SMAD2* being upregulated and *SMAD3* being downregulated as expected. Further, genes involved in cytoskeletal pathways (*FN1, LAMA, ITGB1*) were upregulated in myofibroblasts compared to fibroblasts. Importantly, genes that were previously shown to be changed in asthmatic lungs (*ADAMTS1, DSP, TIMPs, MMPs*) were similarly differentially expressed in myofibroblasts, strongly suggesting that TGF-β mediated differentiation of fibroblasts to myofibroblasts may underlie important changes in the asthmatic airway. We also identified new intermediates of signalling pathways (PKB, PTEN) that are changed in myofibroblasts compared to fibroblasts. We have found a significant number of genes that are altered after TGF-β induced transdifferentiation of WI-38 fibroblasts into myofibroblasts, many of which were expected or predicted. We also identified novel genes and pathways that were affected after TGF-β treatment, suggesting additional pathways are activated during the transition between fibroblasts and myofibroblasts and may contribute to the asthma phenotype.

## Introduction

Asthma is the most common chronic lung disease in children and young adults worldwide and causes a significant burden on health systems as well as loss of productivity^1^. Asthma is a complex disease which is categorised and treated based on clinical characteristics^2^; the variability and lack of treatment response in steroid-resistant allergic asthma suggests this process is suboptimal. A deeper understanding of the molecular/cellular changes in asthma that underpin severity/refractoriness of disease are required to progress toward effective treatment strategies^3^.

Airway structural cells play a crucial role in asthma^3^, expressing inflammatory proteins and releasing mediators^4,5^ that lead to airway remodelling and aberrant responses to external stimuli. Airway remodelling (including increased fibroblasts, myofibroblasts and smooth muscle fibres in airway wall as a result of chronic inflammation) differentiates asthmatic from non-asthmatic airways^6^. The increase in airway fibroblasts and myofibroblasts occurs *via* epithelial to mesenchymal transition (EMT) wherein epithelial cells lose their tight junctions and are transdifferentiated to mesenchymal cells^7,8^ and *via* fibroblast to myofibroblast transition (FMT)^9^, with transforming growth factor (TGF)-β being the central EMT/FMT-inducing cytokine^8,9^. TGF-β is increased in asthmatic airways and further enhanced by infection-induced cytokines (e.g. tumour necrosis factor alpha (TNFα)^10,11^). We have shown that primary airway fibroblasts transdifferentiated to myofibroblasts in the presence of TGF-β have reduced ability to produce type-I interferons (IFNs)^12^. Importantly, glucocorticosteroids (GCS), the mainstay of current asthma treatment, have markedly reduced anti-inflammatory effects on myofibroblasts^13^, which may be at least in part, due to the increased levels of the inactive isoform of glucocorticoid receptor (GRβ) in myofibroblasts^14^. Together, this suggests that increased myofibroblasts that may have differentiated from fibroblasts due to the high levels of TGF-β in the asthmatic airways, may contribute to impaired innate responses that characterise severe, steroid resistant asthma.

In this study we have used the power of unbiased next generation sequencing to delineate the changes in the fibroblast transcriptome induced by TGF-β treatment in the short term and after differentiation into myofibroblasts. As expected, we found that genes that code for intermediates in the TGF-β signalling pathways were differentially expressed after treatment. Also, as expected, genes involved in cytoskeletal pathways were differentially expressed in myofibroblasts compared to fibroblasts. Importantly, several genes that were previously shown to be changed in asthmatic lungs were also differentially expressed in myofibroblasts, strongly suggesting that TGF-β mediated differentiation of fibroblasts to myofibroblasts may underlie important changes in the asthmatic airway. Importantly, our work builds on our previous work and provides further confirmation that the human lung fibroblast WI-38 cell line used in this study represents a good cell culture model to study aspects of asthma after myofibroblast transdifferentiation.

## Methods

### Cells and treatment

WI-38 normal lung fibroblast cells (ATCC) were grown in low-glucose Dulbecco’s Modified Eagle Medium (DMEM) (Sigma) supplemented with 10% foetal bovine serum (FBS) and antibiotics (penicillin, streptomycin, neomycin; Gibco), at 37 °C in an atmosphere of 5% CO_2_. Cells were seeded into T25 flasks and grown to confluence. When confluent, six flasks were treated with TGF-β (2 ng/mL) and the media changed in the remaining flasks (day 0). The following day (day 1) RNA was extracted from 5 treated and 5 untreated flasks. The cells in the remaining flasks (one each of treated and untreated) were used to seed 5 fresh flasks and maintained for 20 days, changing media +/− TGF-β every two days. The number of cells seeded on day 1 was carefully optimised to ensure that flasks would be ~90% confluent on day 20. On day 20 after treatment, RNA was extracted from 5 treated and 5 untreated flasks of cells.

Our previous work^14^ has shown that one day after treatment with TGF-β, WI-38 cells retain their fibroblast phenotype. Clear myofibroblast phenotype (>95% cells) is observed after 20 days of treatment, hence this endpoint (day 20) was used to study changes after transdifferentiation to myofibroblasts.

### Transdifferentiation of WI-38 cells

Transdifferentiation of fibroblasts to myofibroblasts was determined by immunofluorescence assays and western blotting for α-smooth muscle actin (αSMA) and vimentin, as previously demonstrated^14^. Cell expression of αSMA is a specific marker of fibroblast to myofibroblast transdifferentiation, while vimentin is a non-specific fibroblast and myofibroblast marker^15^.

WI-38 cells treated with TGF-β or left untreated were fixed in 4% formaldehyde at days 0, 1, 10 or 20 for 10 min and permeabilized with 0.5% Triton X-100 for 10 min. Cells were then incubated with antibodies to vimentin and αSMA (1:100) for 30 min at room temperature and bound antibodies detected by Alexa Fluor conjugated secondary antibodies (1:1000, 30 min; Life Technologies). Coverslips were mounted on slides in ProLong Gold reagent with DAPI (Life Technologies). Samples were examined under a Nikon Ti Eclipse confocal laser-scanning microscope (CLSM) with Nikon 60×/1.40 oil immersion lens (Plan Apo VC OFN25 DIC N2; optical section of 0.5 µm) and the NIS Elements AR software (Nikon Corporation, Japan).

WI-38 cells treated with TGF-β or left untreated, were lysed in RIPA lysis buffer (150 mM NaCl, 1% Triton X-100, 0.5% sodium deoxycholate, 0.1% SDS, 50 mM Tris, with protease and phosphatase inhibitors) at day 1 or day 20. Lysates were electrophoresed on 10% SDS-PAGE and proteins transferred to nitrocellulose membranes. Cell lysates were subjected to SDS-polyacrylamide electrophoresis followed by transfer to nitrocellulose membranes in Tris-glycine-ethanol buffer (25 mM Tris HCl, 192 mM glycine, 20% ethanol). Blots were blocked for 1 h in Odyssey Blocking Buffer containing 0.1% Tween 20, prior to overnight incubation with primary antibodies (αSMA, ab7817, Abcam and vimentin, sc-5565, Santa Cruz Biotechnology) diluted in Odyssey Blocking Buffer containing 0.1% Tween 20 at 4 °C. Primary antibodies were detected using LI-COR IRDye Infrared Dye (1:15000) secondary antibodies. Blots were visualized using the Odyssey Fc Infrared Imager (LI-COR Biotechnology, NE, USA). Blots were also probed for expression of vinculin, ADAMTS1, SMAD7, RHEB, with tubulin being used as loading control.

### RNA extraction

RNA samples were collected on day 1 and day 20 after TGF-β treatment and 5 replicates were collected for treated/untreated samples at both time points. Media was removed from the flasks and cells washed with cold PBS. 1 mL of TRIzol was added to each flask to cover the cell monolayer, cells were scraped from the flask, transferred to a microcentrifuge tube and incubated at room temperature for 5 min. Next, 200 µL of chloroform was added and the samples vortexed for 15 sec, followed by incubation on ice for 2–3 min and centrifugation at 10,000 rpm for 15 min at 4 °C. The aqueous phase was removed into a new tube and an equal volume (~500 µL) of isopropanol was added. Samples were vortexed briefly then placed at −20 °C to allow RNA to precipitate overnight. The following day, samples were centrifuged at 10,000 rpm for 10 min at 4 °C. The supernatant was removed, and the RNA pellet was washed in 1 mL of 75% ethanol in DEPC water by centrifugation at 7500 rpm for 5 min at 4 °C. The pellet was air dried to remove residual ethanol and 30–50 µL of DEPC water was added, depending on pellet size. RNA quality was assessed using a Nanodrop instrument and Bioanalyzer 2100 (Agilent) and samples stored at −80 °C until required.

### RNA sequencing

Three RNA samples for each timepoint and treatment (12 samples in total) were selected based on quantity and quality of the collected RNA (RIN >9.5). The libraries were generated using the New England Biolabs Ultra Directional RNA library Prep Kit for Illumina following the manufacturer’s recommended protocol using 1 µg of total RNA. Library size distributions and concentrations were assessed using the Bioanalyzer 2100. Libraries were pooled in equimolar amounts to a final concentration of 2 ng/µL and stored at −20 °C until required. Sequencing was performed on an Illumina NextSeq. 500 using a Mid Output paired end format kit at the Ramaciotti Centre for Genomics, University of New South Wales.

### cDNA synthesis and real-time PCR

cDNA was synthesised using the High Capacity cDNA reverse transcription kit (Applied Biosystems) following the manufacturer’s instructions. Briefly, a 2x master mix was prepared containing 2x reverse transcription buffer, 8 mM dNTPs, 2x RT random primers and 50U MultiScribe reverse transcriptase. 10 µL of the 2x master mix was pipetted into each well. 1 µg of RNA was diluted to a final volume of 10 µL in DEPC water, then added to the master mix, to give a final volume of 20 µL. The reverse transcription reaction was performed using the following conditions: 25 °C for 10 min, 37 °C for 120 min, 85 °C for 5 min then hold at 4 °C. After cycling, 30 µL of DEPC water was added to each sample, and cDNA was stored at −20 °C until required.

To generate a standard curve for real-time PCR analysis, an equal volume from each sample was pooled. Standard 1 was generated by diluting the initial pool 1:10. The cDNA was then serially diluted 1:5 to generate standards 2–5. Samples for testing were diluted 1:50 prior to analysis. Real-time PCR reactions were performed using SsoAdvanced SYBR green supermix (Biorad). Mastermix containing SsoAdvanced SYBR green, forward and reverse primers to a final concentration of 300 nM and water to 16 µL was pipetted into each well. 4 µL of standard, sample or water was added to the appropriate wells. Plates were sealed, centrifuged at 1000 rpm for 1 min then cycled on a Biorad CFX96 using the following conditions: 98 °C for 30 sec, 40 cycles of 95 °C for 10 sec, 60 °C for 30 sec followed by a plate read, then one cycle of 95 °C for 10 sec followed by a melt curve from 60 °C to 95 °C at 0.5 °C increments with 0.5 sec dwell time and plate read.

All samples were amplified in duplicate and GAPDH was included as a loading control. Data were analysed using CFX Manager v3.1 (Bio-Rad), and are presented in arbitrary units relative to GAPDH. Primers were purchased from Sigma and the sequences are available on request.

### Data analysis

The resulting raw sequence data reads from RNA sequencing were trimmed using the Trimmomatic tool^16^ to ensure the highest quality sequences for mapping and differential expression analysis. The remaining high quality reads were mapped to the human reference genome (hg19) using the R package Rsubread^17^. Genes having less than 10 count-per-million reads (cpm) in half of the 48 samples were filtered out using the “edgeR” library. The R package limma^18,19^ was used for differential gene expression analysis, with multiple testing adjustments made using the Benjamini and Hochberg method^20^. Significant differentially expressed genes for a particular comparison were identified by selecting genes with a p-value < 0.01. Pathway analysis was performed using GeneGo.

GraphPad Prism v7 was used to assess differences in expression in real-time PCR results for different conditions. Statistical analysis was performed using two-way ANOVA followed by correction for multiple comparisons using Tukey’s test. Data were then grouped by treatment and time. Volcano plots of the data were generated with XLStat within Excel. Correlation between the RNASeq data and that obtained from real-time PCR for the same set of genes was assessed by XLStat within Excel and is represented as R^2^, the square of the Pearson’s correlation coefficient.

## Results

### TGF-β induced transdifferentiation

**Figure 1:**
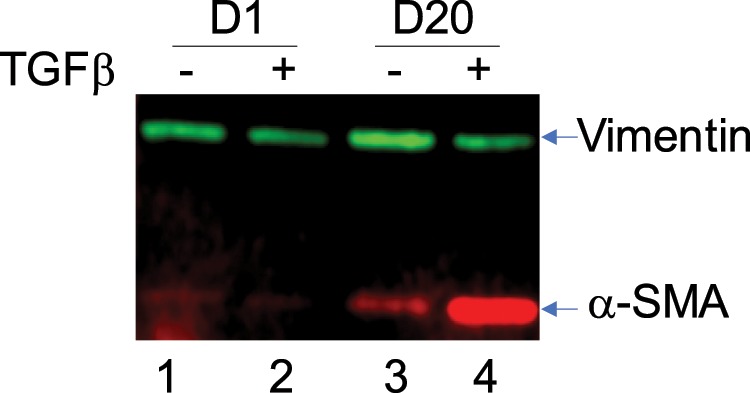
Culture of WI-38 cells in presence of TGF-β for 20 days leads to transdifferentiation. WI-38 cells were cultured in presence of TGF–β (2 ng/ml, indicated by ‘+’ above the lane) or left untreated (−) for 20 days. Cells were lysed on day 1 and day 20 and analysed for expression of vimentin and αSMA by western blotting as described in the Methods section. The specific bands are indicated.

As we have shown previously^14^, TGF-β treatment of WI-38 fibroblast cell line over 20 days resulted in differentiation of the cell line to a myofibroblast phenotype as shown by the increased expression of αSMA (Fig. 1) compared to cells without TGF-β (compare lower band in lanes 3 and 4). Culture of WI-38 cells over 20 days with TGF-β had no effect on vimentin expression (compare higher band in lanes 2 and 4). The image of the full-length blot is provided in Supplementary Information (Fig. S1). Consistent with the western blot analysis and as we have shown previously^14^, indirect immunofluorescence showed that TGF-β treatment for 20 days induced fibroblast to myofibroblast transdifferentiation in WI-38 cells, indicated by the presence of positive αSMA staining in majority of the treated cells (Fig. S2, images in the middle column). No WI-38 cells were positive for αSMA after treatment for one day (images labelled Day 1) and only rare cells after treatment for 10 days (images labelled Day 10). WI-38 cells were positive for vimentin at all time points analysed (day 0, 1, 10, 20).

### Differential gene expression summary

**Figure 2:**
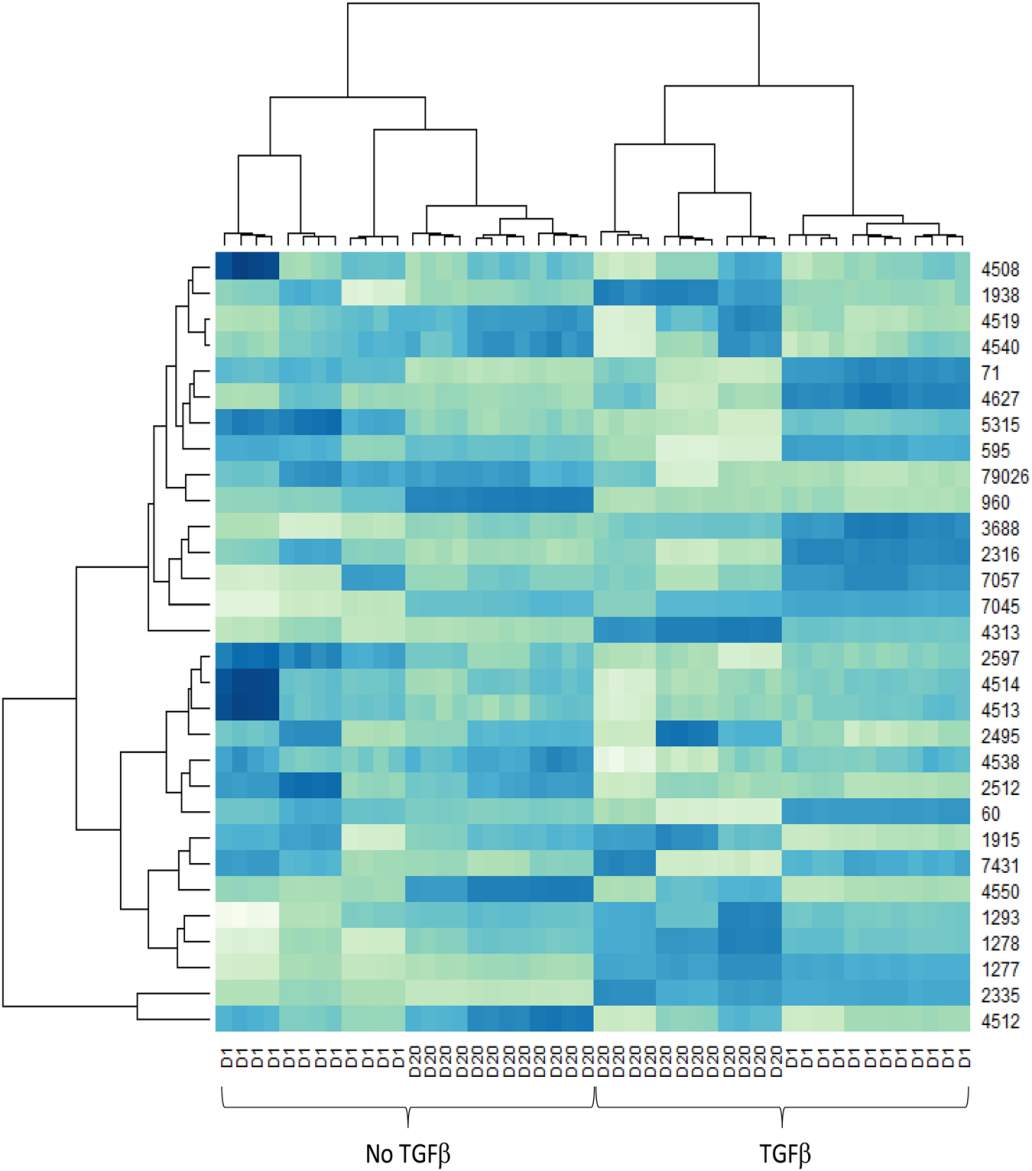
RNASeq samples cluster by treatment and timepoint. Hierarchical clustering of each sample was performed using the 30 most highly expressed genes. Samples with and without TGF-β are indicated (TGFβ, no TGFβ). D1 – day one after treatment, D20 – day 20 after treatment. Numbers on the right are gene ID numbers as found in the NCBI Gene database. Gradient from light to dark indicates the gene expression value, where dark blue represents higher expression and light green represents lower expression.

**Figure 3:**
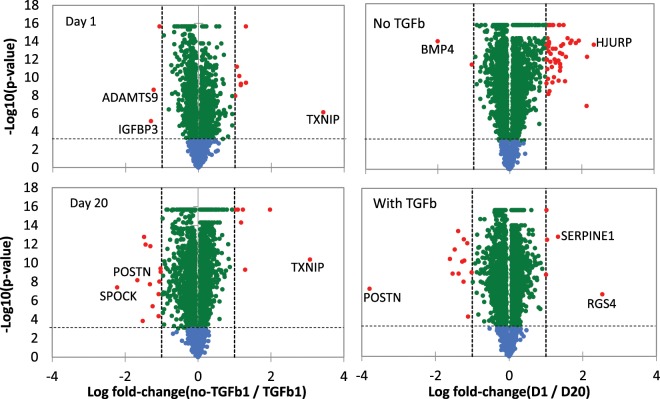
Volcano plots of RNASeq samples by treatment and timepoint. The negative log10 transformed p-values are plotted against log2 fold change. The comparisons are log fold change in no TGF-β vs TGF-β for day and day 20 of culture (plots on the left) and in day 1 vs day 20 without (no TGF-β) or with TGF-β (plots on the right). Log2 fold change = ±1 is indicated by dashed vertical lines. The horizontal dashed lines indicate p = 0.001. Blue dots indicate genes with p > 0.001, green dots indicate genes with p < 0.001, red dots indicate genes with p < 0.001, log2 fold change larger than ±1.0. Selected differentially expressed genes are labelled.

Following quality control and mapping for all samples and prior to differential gene expression analysis, hierarchical clustering was performed using a matrix of Euclidean distances calculated from the mapped read counts for the 30 most highly expressed genes. The data in Fig. 2 show that the samples cluster primarily according to the time point and treatment, as expected. Volcano plots of the data showed that limited number of genes were significantly up or down regulated, with majority of the changes remaining within ±1 log fold change (Fig. 3).

Differentially expressed genes were determined for the following comparisons: Day 1 with TGF-β versus D1 without TGF-β (D1+ TGF-β vs D1) – to detect gene expression changes that occur early after initial short term TGF-β treatment; Day 20 with TGF-β versus D20 without TGF-β (D20+ TGF-β vs D20) – to detect gene expression changes that occur over long term TGF-β treatment but that are not related to any changes that occur due to the length of time cells are being cultured; Day 20 with TGF-β versus D1 with TGF-β (D20+ TGF-β vs D1+ TGF-β) – to detect gene expression changes that occur after long term TGF-β treatment, above what may occur after short term treatment; Day 20 without TGF-β versus Day 1 without TGF-β (D20 vs D1) – to detect gene expression changes that occur due to cells being in culture long term. Using an adjusted p value (q value) cut off p < 0.01, and log2(fold change) <−2 or >2, 34 genes were identified in the D1+ TGF-β vs D1 comparison; 61 genes were identified in the D20+ TGF-β vs D20 comparison; 49 genes were identified in the D20+ TGF-β vs D1+ TGF-β comparison; and 126 genes were identified in the D20 vs D1 comparison. The top 10 differentially expressed genes (sorted by most upregulated to most downregulated) for each comparison are shown in Table 1.

**Table 1:** Top 10 differentially expressed genes for each of the four treatment comparisons.

D1+ TGFβ vs D1	logFC	D20+ TGFβ vs D20	logFC	D20+ TGFβ vs D1+ TGFβ	logFC	D20 vs D1	logFC
IGFBP3	4.15	TXNIP	−5.18	RASL12	3.75	HJURP	−3.42
TXNIP	−3.57	A2M	−4.70	ADAMTS5	3.64	TTK	−3.32
AMIGO2	3.53	ADAMTS19	−4.13	ADAMTS9	3.62	SKA1	−3.30
ADAMTS9	−2.73	LZTS1	−3.53	RGS4	−3.32	AURKB	−3.28
HSD17B2	−2.59	SPOCK1	3.45	FAM43A	3.05	BMP4	3.27
SEMA7A	2.57	GPX3	−3.41	ALDH1A3	2.91	KIF18B	−3.27
DLL4	−2.54	POSTN	3.33	MXRA5	2.88	SKA3	−3.21
NPTX1	−2.52	ARRDC4	−3.04	SLC40A1	2.84	NEK2	−3.21
RASL12	−2.52	ANKRD33B	−2.92	SLC7A14	2.73	CDC20	−3.21
SERPINE2	2.42	WFDC1	−2.86	SERPINE1	−2.72	MKI67	−3.17

### Pathway analysis reveals pathways altered after TGF-β treatment

Specific pathways that were altered by TGF-β treatment were identified by pathway analysis. The top pathway maps for the D20+ TGF-β vs D20 comparison, representing long term TGF-β-initiated changes in gene expression, included cytoskeleton remodelling (including TGF-β and wingless-type mouse mammary tumour virus (*WNT*) remodelling), cell adhesion pathways (including chemokines and adhesion, and integrin-mediated cell adhesion and migration), the epidermal growth factor receptor (*EGFR*) signalling pathway and regulation of eukaryotic initiation factor 4 F (*EIF4F*) activity. The top 10 pathways are shown in Table 2.

**Table 2:** Top 10 pathways for D20+ TGFβ vs D20 samples.

	Pathway Maps	Total # genes	# genes in data	pValue
1	Cytoskeleton remodeling_TGF, WNT and cytoskeletal remodeling	111	74	3.934E-29
2	Cytoskeleton remodeling_Cytoskeleton remodeling	102	67	6.556E-26
3	Cell adhesion_Chemokines and adhesion	100	58	9.719E-19
4	Immune response_Oncostatin M signaling via MAPK in human cells	37	29	3.477E-15
5	Immune response_Oncostatin M signaling via MAPK in mouse cells	35	27	6.413E-14
6	Development_EGFR signaling pathway	71	41	1.523E-13
7	Cell cycle_The metaphase checkpoint	36	27	2.115E-13
8	Cell adhesion_Integrin-mediated cell adhesion and migration	48	32	2.588E-13
9	Cell adhesion_Role of tetraspanins in the integrin-mediated cell adhesion	37	27	6.455E-13
10	Translation_Regulation of EIF4F activity	53	33	1.942E-12

The top pathway maps for D20+ TGF-β vs D1+ TGF-β comparison, to detect long term changes in response to TGF-β treatment, above what is seen after the initial 24 hours of treatment, included cytoskeleton remodelling (including TGF-β and *WNT* remodelling), cell adhesion pathways (including chemokines and adhesion, and integrin-mediated cell adhesion and migration) regulation of *EIF4F* activity and intermediates in the signalling pathways for protein kinase B (PKB) and phosphatase and tensin homolog (PTEN). The top 10 pathways are shown in Table 3.

**Table 3:** Top 10 pathways for D20+ TGFβ vs D1+ TGFβ samples.

	Pathway Maps	Total # genes	# genes in data	pValue
1	Cytoskeleton remodeling_TGF, WNT and cytoskeletal remodelling	111	81	6.586E-31
2	Cytoskeleton remodeling_Cytoskeleton remodeling	102	74	4.251E-28
3	Cell adhesion_Chemokines and adhesion	100	62	7.474E-18
4	Transport_Clathrin-coated vesicle cycle	71	45	2.746E-13
5	Cell adhesion_Integrin-mediated cell adhesion and migration	48	35	4.660E-13
6	Translation_Regulation of EIF4F activity	53	37	5.199E-13
7	Cell cycle_Influence of Ras and Rho proteins on G1/S Transition	53	37	5.199E-13
8	Signal transduction_AKT signaling	43	32	1.766E-12
9	Signal transduction_PTEN pathway	46	33	3.282E-12
10	Development_WNT signaling pathway. Part 2	53	36	3.282E-12

### RNASeq analysis is confirmed by real-time PCR

We next chose a series of targets within the identified pathways for confirmation testing. A number of genes were identified in multiple pathways, and details of the genes found in each pathway as identified in the initial RNASeq analysis are shown in Table 4. The target list included genes that showed significant differences in expression between D20 and D20+ TGF-β, as the main focus of this study was to identify changes that occur after TGF-β induced transdifferentiation. Details of the log fold change and adjusted p-values of comparisons between D20 +TGF-β vs D20 for genes included in the confirmation testing are shown in Table 5. Details of the log fold change and adjusted p-values of comparisons between D20+ TGF-β vs D1+ TGF-β for genes included in the confirmation testing are shown in Table 6.

**Table 4:** Biological pathways in which gene expression is significantly altered with TGF-β treatment.

Biological pathway	Genes from this study in pathway
Translation_Regulation of EIF4F activity	RHEB
Cytoskeleton remodeling_TGF, WNT and cytoskeletal remodeling	FN1, VCL, RHEB, LAMA1, CSNK2B, CDKN2B, SMAD2, SMAD3
Cell adhesion_Chemokines and adhesion	ITGB1, FN1, VCL, LAMA1, LAMA4, MMP1
Cell adhesion_Integrin-mediated cell adhesion and migration	ITGB1, FN1, VCL, LAMA1
Cell adhesion_Role of tetraspanins in the integrin-mediated cell adhesion	ITGB1, FN1, VCL
Immune response_Oncostatin M signaling via MAPK in human cells	LDLR, TIMP1
Signal transduction_AKT signaling	RHEB
Signal transduction_PTEN pathway	ITGB1, RHEB

**Table 5:** Data summary from RNASeq analysis – D20+ TGFβ vs D20.

Gene Symbol	Gene Name	LogFC	Adjusted p value
**Up-regulated genes**			
CDKN2B	cyclin-dependent kinase inhibitor 2B (p15, inhibits CDK4)	2.424	5.22E-26
FN1	fibronectin 1	1.956	6.05E-36
LAMA1	laminin, alpha 1	1.117	2.50E-24
MMP1	matrix metallopeptidase 1	2.436	3.49E-27
**Down-regulated genes**			
LAMA4	laminin, alpha 4	−1.698	4.33E-31
SMAD3	SMAD family member 3	−1.849	6.41E-28

**Table 6:** Data summary from RNASeq analysis – D20+ TGFβ vs D1+ TGFβ.

Gene Symbol	Gene Name	LogFC	Adjusted p value
CDKN2B	cyclin-dependent kinase inhibitor 2B (p15, inhibits CDK4)	1.009	8.38E-15
FN1	fibronectin 1	0.203	0.001
LAMA1	laminin, alpha 1	0.398	7.28E-09
MMP1	matrix metallopeptidase 1	1.443	6.15E-18
LAMA4	laminin, alpha 4	−0.554	5.63E-11
SMAD3	SMAD family member 3	—	—

**Figure 4:**
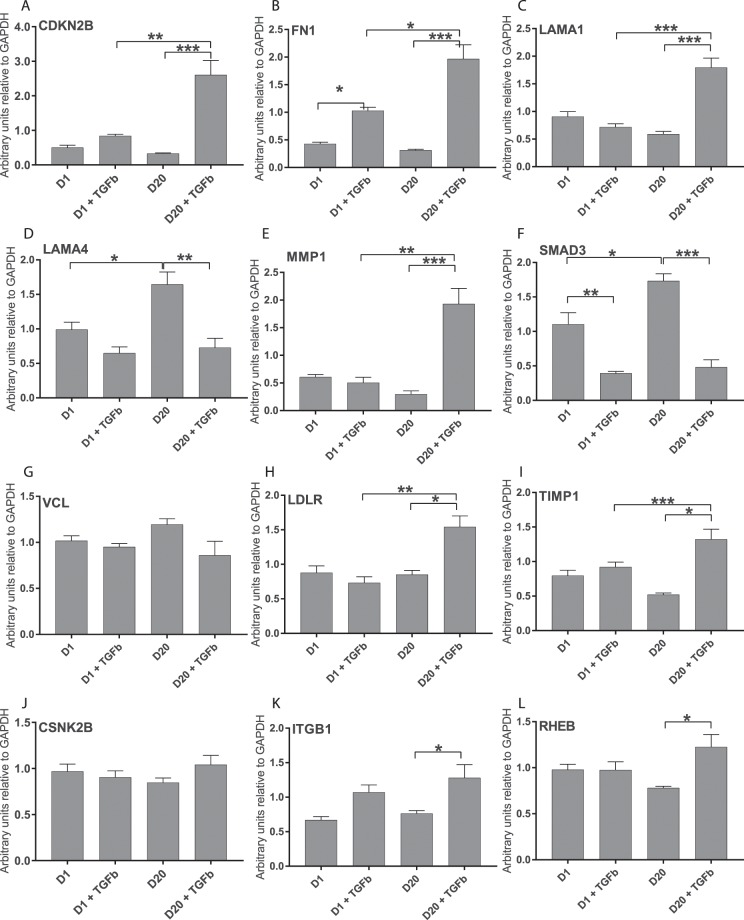
Real-time PCR confirmation of RNASeq results. Real-time PCR was performed on 5 samples for each of the treatment times, Day 1 (D1), D1+ TGFβ, D20 and D20+ TGFβ for a series of genes. Graphs show the number of arbitrary units relative to GAPDH expression for: (**A**) CDKN2B, (**B**) FN1, (**C**) LAMA1, (**D**) LAMA4, (**E**) MMP1, (**F**) SMAD3, (**G**) VCL, (**H**) LDLR, **(I**) TIMP1, (**J**) CSNK2B, (**K**) ITGB1, **(L**) RHEB. Mean ± SEM are shown for each set, *p < 0.05, **p < 0.01, ***p < 0.001.

For each target gene shown in Table 4, all 5 replicate RNA samples were included in the real-time PCR analysis and samples were analysed in duplicate. Data for each gene are shown in Fig. 4 and represented relative to GAPDH. GAPDH was chosen as reference as analysis of RNASeq data showed that GAPDH was stably expressed across replicates and at different time points (Fig. S3). We analysed the genes for differences in expression between D20+ TGF-β and D1+ TGF-β and compared the real-time PCR results (Fig. 4) to the RNASeq results (Table 5).

The first seven genes (cyclin-dependent kinase 4 inhibitor B (CDKN2B), fibronectin 1 (*FN1*), laminin subunit alpha 1 (*LAMA1*), laminin subunit alpha 4 (*LAMA4*), matrix metalloproteinase 1 (*MMP1*), *SMAD3* (Smad proteins are homologues of the Drosophila protein, mothers against decapentaplegic (*Mad*) and the *Caenorhabditis elegans* protein Sma), vinculin (*VCL*)) showed highly significant differences (p < 1 × 10^−20^) in gene expression between D20+ TGF-β and D20 samples, as detected by RNASeq analysis. *CDKN2B*, *FN1*, *LAMA1* and *MMP1* were upregulated, while *LAMA4*, *SMAD3* and *VCL* were downregulated in D20+ TGF-β vs D20 samples. The real-time PCR results confirmed a significant difference (p < 0.005) in gene expression between D20+ TGF-β and D20 samples for *CDKN2B*, *FN1*, *LAMA1*, *LAMA4*, *MMP1* and *SMAD3* (Fig. 4A–F); the direction of the fold change was maintained. No significant difference in *VCL* gene expression was detected in the confirmation real-time PCR (Fig. 4G), however the direction of the fold change (D20+ TGF-β had lower levels of RNA compared to D20) was maintained.

The next 2 genes (low density lipoprotein receptor (*LDLR*) and tissue inhibitor of metalloproteinases metallopeptidase inhibitor 1 (*TIMP1*) showed moderately significant upregulation (p < 1 × 10^−5^) in gene expression between D20+ TGF-β and D20 samples, as detected by RNASeq analysis and a significant upregulation at D20+ TGF-β vs D20 by real-time PCR (Fig. 4H,I).

The next 3 genes (casein kinase II subunit beta (*CSNK2B*), integrin beta 1 (*ITGB1*) and Ras homolog enriched in brain (*RHEB*)) were included as they were identified in pathways of interest. All three genes showed low levels of significant differences (p < 1 × 10^−3^) in gene expression between D20+ TGF-β and D20 samples, as detected by RNASeq analysis. *CSNK2B* was downregulated, while *ITGB1* and *RHEB* were upregulated in D20+ TGF-β vs D20 samples. No significant difference in gene expression between D20+ TGF-β and D20 samples was identified for *CSNK2B*, however a significant upregulation was seen for *RHEB* and *ITGB1* (Fig. 4J–L).

We next analysed the same cohort of genes for any differences in expression between D20+ TGF-β and D1+ TGF-β and compared the real-time PCR results (Fig. 4) to the RNASeq results (Table 6). There was only one gene, *VCL*, that showed a highly significant difference (p < 1 × 10^−20^) in gene expression between D20+ TGF-β and D1+ TGF-β samples in the RNASeq data, though the logFC detected by RNASeq was small at −0.483. The real-time PCR results show no significant difference in expression between these samples for *VCL* (Fig. 4G).

Of the genes demonstrating moderately significant differences (p < 1 × 10^−5^) in gene expression between D20+ TGF-β and D1+ TGF-β in the RNASeq data, namely *CDKN2B, LAMA1, LAMA4, MMP1, LDLR, CSNK2B* and *ITGB1*, only *CDKN2B*, *LAMA1*, *MMP1* and *LDLR* showed a significant difference in these samples by real-time PCR (Fig. 4A,C,E,H). The remaining genes, *LAMA4*, *CSNK2B*, and *ITGB1*, showed no significant difference between D20+ TGF-β and D1+ TGF-β samples. There were two genes that showed a low level of significance (p < 1 × 10^−3^) between D20+ TGF-β and D1+ TGF-β samples in the RNASeq analysis, *FN1* and *RHEB*. Of these genes the real-time PCR detected a significant difference in gene expression only for *FN1* (Fig. 4B).

There were two genes included in the real-time PCR analysis that were not identified as having any level of significant difference in expression between D20+ TGF-β and D1+ TGF-β in the RNASeq analysis, *SMAD3* and *TIMP1*, and no difference in expression was detected for *SMAD3* by real-time PCR (Fig. 4F,I). However, a highly significant upregulation in expression was detected at D20+ TGF-β compared to D1+ TGF-β for *TIMP1*.

**Figure 5:**
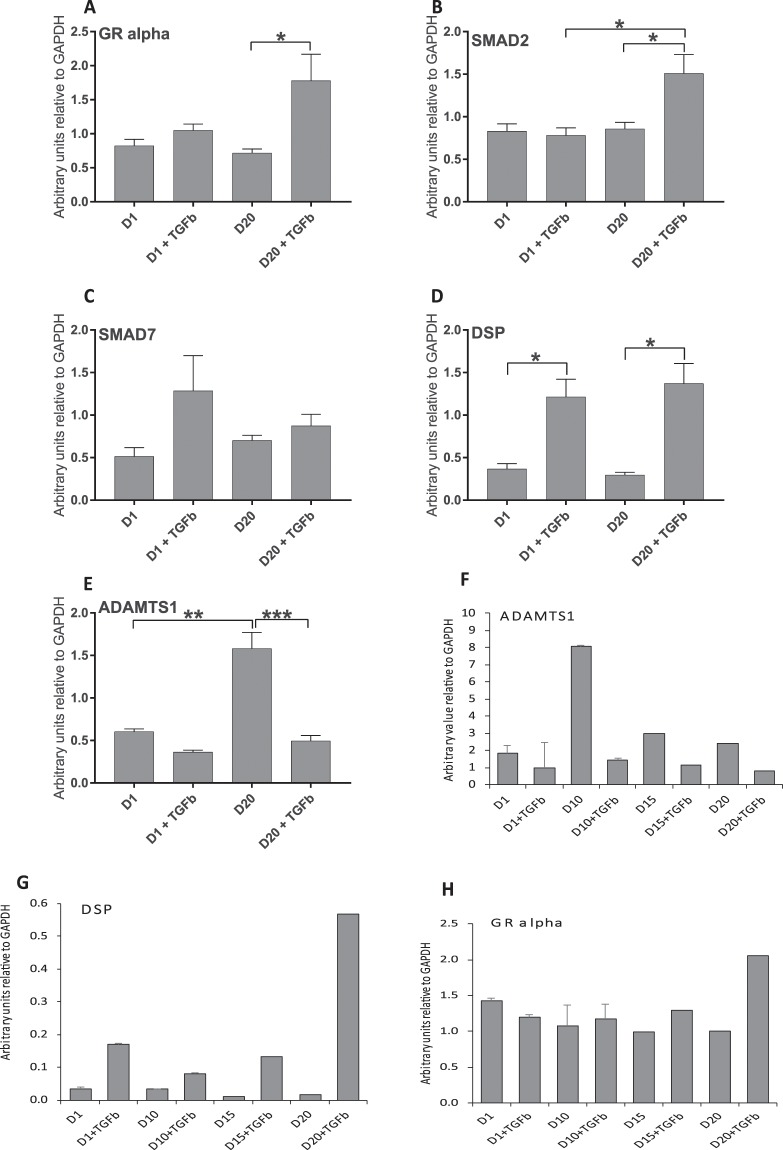
Real-time PCR of asthma and TGF-β pathway genes. (**A–E)** Realtime PCR was performed on as per the legend to Fig. 4 for a series of genes identified from literature to be important in asthma. Graphs show the number of arbitrary units relative to GAPDH expression for: (**A**) GR-alpha, (**B**) SMAD2, (**C**) SMAD7, (**D**) DSP, (**E**) ADAMTS1. (**F–H)** Real-time PCR was performed as in (**A–E**) above for treatment times D1, D1+ TGFβ, D10, D10+ TGFβ, D15, D15+ TGFβ, D20 and D20+ TGFβ for (**F**) ADAMTS1, (**G**) DSP, (**H**) GR-alpha. Mean ± SEM are shown for each set, *p < 0.05, **p < 0.01, ***p < 0.001.

We then selected a number of genes that were of interest based on the literature for relevance to asthma (A disintegrin and metalloproteinase with thrombospondin motifs 1 (*ADAMTS1*)^21^, desmoplakin (*DSP*)^22^ and glucocorticoid receptor (*GR*)^23^) or for relevance in TGF-β pathways (*SMAD2* and *SMAD7*)^24^ and analysed the samples by real-time PCR (Fig. 5). We found a significant difference in gene expression between D20+ TGF-β and D20 samples for all genes except for *SMAD7* (Fig. 5A–E). A targeted search for these genes in the RNASeq data found that *ADAMTS1* was significantly downregulated, while *DSP* and *GR* were significantly upregulated in D20+ TGF-β vs D20, and *SMAD2* was significantly upregulated in D20+ TGF-β versus D1+ TGF-β samples (Table 7); this data correlated well with the real-time PCR results as shown by correlation analysis (Fig. S4, R2 = 0.7534).

**Table 7:** RNASeq results for genes selected based on literature.

Dataset	Gene Symbol	Gene Name	LogFC	Adjusted p value
D20+ TGFβ vs D20	ADAMTS1	ADAM metallopeptidase with thrombospondin type 1 motif, 1	−2.210	1.42E-27
	DSP	desmoplakin	1.413	3.29E-13
	SMAD2	SMAD family member 2	—	—
	SMAD7	SMAD family member 7	—	—
	NR3C1/GR	nuclear receptor subfamily 3, group C, member 1 (glucocorticoid receptor)	0.423	1.43E-08
D20+ TGFβ vs D1+ TGFβ	ADAMTS1	ADAM metallopeptidase with thrombospondin type 1 motif, 1	—	—
	DSP	desmoplakin	—	—
	SMAD2	SMAD family member 2	0.115	0.006
	SMAD7	SMAD family member 7		
	NR3C1/GR	nuclear receptor subfamily 3, group C, member 1 (glucocorticoid receptor)	—	—

We extended our real-time PCR analysis for *ADAMTS1, DSP* and *GR* to determine at which point in the transdifferentiation the observed change in gene expression became evident (Fig. 5F–H). We found that *ADAMTS1* was upregulated after 10 days in culture (D10); this change was suppressed in TGF-β treated samples (Fig. 5F, compare TGF-β treated with untreated samples at D1, D10, D15 and D20). *DSP* was upregulated on D1 in TGF-β treated versus untreated samples and this trend continued through the experiment, with a significant change observed at D20 (Fig. 5G). The data strongly suggests that TGF-β induced pathways suppress the expression of *ADAMTS1* with or without transdifferentiation, while the upregulation of *DSP* is directly associated with the myofibroblast phenotype. The expression of *GR* (specifically *GR-alpha*) was increased at D20 with TGF-β treatment compared to the untreated samples at D20, with no significant change in intermediate days, indicating that changes in *GR-alpha* expression are a characteristic of the myofibroblast phenotype.

Overall, these data demonstrate the different pathways involved in the transdifferentiation of fibroblasts to myofibroblasts, highlighting known intermediates in well studied pathways such as cytoskeleton remodelling via TGF-β pathways as well as identifying new intermediates in the PKB and PTEN signal transduction pathways that are gaining importance in transdifferentiation. Of the genes selected for real-time PCR confirmation, majority demonstrated the same significant changes in gene expression between the different samples, or if no significant differences were observed, the shift in gene expression was always in the same direction.

### TGF-β treatment leads to reduced SMAD7 and increased RHEB protein

**Figure 6:**
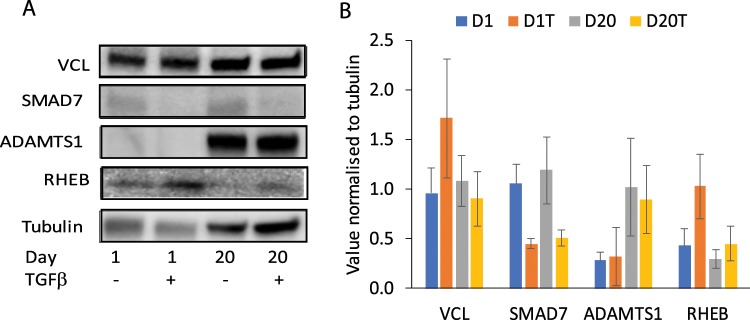
Western blot analysis of selected proteins. Western blot analysis was performed on whole cell lysates for each of the treatment times, D1, D1+ TGFβ, D20 and D20+ TGFβ for VCL, ADAMTS1, RHEB, SMAD7 with tubulin used as loading control. Data represent two independent experiments. (**A**) blots showing the staining for individual proteins indicated on the left, time and treatment are indicated below the blots. (**B**) histogram showing the densitometric intensity of each band for the indicated proteins normalised to its cognate tubulin band; data are mean ± SEM. D1 – day 1 without treatment, D1T – day 1 with treatment, D20 – day 20 without treatment, D20T – day 20 with treatment.

We chose four gene products to study whether the observed gene expression changes by RNASeq and real-time PCR resulted in changes in the expression levels of the proteins encoded by these genes; tubulin was used as a loading control (Fig. 6A,B). A TGF-β associated reduction in SMAD7 was observed both at D1 and after transdifferentiation at D20, while a trend towards increased RHEB in the presence of TGF-β compared to its absence, was observed both at D1 and at D20; no change was observed in VCL levels. These data are in agreement with the RNASeq and real-time PCR data. Culture over 20 days resulted in increased ADAMTS1, which was not changed in the presence of TGF-β. ADAMTS1 is a secretory protein and we may not be able to assess total changes in protein expression in the current experimental design. We have previously shown^14^ that SMAD3 is reduced in TGF-β treated WI-38 cells, correlating with our current RNASeq and real-time PCR data. A trend towards increase of SMAD2 was observed in myofibroblasts^14^ while short term treatment with TGF-β resulted in reduced expression, completely in agreement with our current real-time PCR and RNASeq data.

## Discussion

In the current study, we show that a large number of genes are modulated in an airway fibroblast cell line in response to TGF-β treatment and on transdifferentiation to myofibroblasts^25^. As expected, expression of intermediates in pathways that regulate cytoskeleton and focal adhesions was changed as well as expression of intermediates in TGF-β signalling pathways. Importantly, expression of several genes known to have a role in asthma was changed, directly corresponding to the changes observed in asthma. The data presented here builds on our previous work^14^ and strongly suggests the feasibility of using TGF-β treated WI-38 cell line as a cell culture model to study the role of myofibroblasts in asthma.

In the context of the asthmatic airway, TGF-β released by the destruction of epithelial cells^26^ can lead to transdifferentiation of fibroblasts to myofibroblasts that can contribute to airway remodelling via production of specific growth factors, extracellular matrix (ECM) modulating proteins and cytokines^27,28^. Fibroblasts and myofibroblasts are known to be increased in the remodelled airways in severe asthma^6,29^, and increasing evidence supports an important role for these cells in the clinical symptoms of asthma.

We have previously shown that TGF-β treatment of an airway fibroblast cell line induces fibroblast to myofibroblast transdifferentiation accompanied by an altered TGF-β signalling pathway, with decreased Smad3 expression, and impact on glucocorticoid responses due to changes in the expression of the GR isoforms^14^.

The main focus of our current study was to investigate the role that myofibroblasts may play in the clinical symptoms of asthma, thus extending and adding to our previous study^14^. Our data confirm our previous finding that prolonged, heightened TGF-β levels can cause transdifferentiation of fibroblasts. Additionally, the resultant myofibroblasts have a gene expression profile that closely matches the observed expression profile from asthmatic airways.

Of the top 10 differentially expressed genes for each of the four comparisons, 50% had been identified as altered in asthma or airway inflammation previously^30–40^. Most of the genes differentially altered in D1+ TGF-β vs D1 (6/10^30–35^) and D20+ TGF-β vs D20 (7/10^36–38,40^) have been described previously, while half of those altered in D20+ TGF-β vs D1+ TGF-β^32,38,39^ and only 2 in D20 vs D1 comparison^41,42^ had been previously described in asthma, airway remodelling or airway inflammation.

*CDKN2B* (cyclin-dependent kinase inhibitor 2B) was significantly increased in myofibroblasts compared to fibroblasts. This was expected as the expression of CDKN2B is known to be significantly induced by TGF-β in other tissue systems^43^. A major characteristic of airway remodelling is the increase in ECM components, leading to ‘stiffening’ of airways^44,45^. We found that major factors known to be involved in increased ECM thickness were upregulated in response to TGF-β along with factors that breakdown excess ECM. *FN1* (fibronectin 1) was significantly increased in myofibroblasts compared to fibroblasts. Fibronectin is important in cell adhesion and migration processes including embryogenesis, wound healing, and metastasis. Importantly, fibronectin deposition in the airway wall is increased in asthma^46–48^ and our data suggest that myofibroblasts can contribute to this effect^49^.

The expression of *LAMA1* and *LAMA4* was changed, with the former being upregulated and the latter being downregulated in myofibroblasts. The laminins are a major component of the basement membrane and implicated in a wide variety of biological processes including cell adhesion, differentiation, migration, signalling, neurite outgrowth and metastasis^50^. The increase in *LAMA1* expression was expected given its known functions in the ECM. Importantly, LAMA1 expression is increased in animal models of airway remodelling^51^ and bronchial biopsies from patients with severe asthma^52^. Our data suggests that increased myofibroblasts may, at least in part, be responsible for this increase. At this time, we do not know the significance of the downregulation of *LAMA4*, which could suggest either that laminins self-regulate each other or that different laminins have opposing roles in airway remodelling.

Myofibroblasts are known to contribute to the contractile behaviour of the airways via their expression of molecules that directly interact with and impact on the ECM and the cytoskeletal network^53^. We found that focal adhesion molecule *ITGB1* was upregulated in myofibroblasts as expected^54–56^. *ITGB1* encodes an integrin beta subunit that functions as receptor for fibronectin; along with the increased expression of fibronectin, this would result in enhanced functional consequences for the airway. Interestingly, a recent study in a guinea pig model found that ITGB1 expression was increased in asthma^57^. Vinculin (*VCL*), that has a role in regulating cell–matrix adhesion, including the assembly, turnover, and strength of focal adhesions, as well as the transmission of force by these cellular structures, was downregulated in myofibroblasts. This finding is in contrast to findings of increased VCL in asthma models^58^, and suggests that TGF-β transdifferentiated myofibroblasts do not contribute to this aspect of the asthma phenotype.

Expression of matrix metalloproteases (MMPs) is increased in asthmatic airways and these enzymes play a central role in the pathology of asthma^59,60^. *MMP1* was upregulated in myofibroblasts, suggesting a potential source of the increased enzyme in the airway. A recent study has shown that MMP1 is associated with bronchial hyperresponsiveness and asthma exacerbation severity^59^.

*TIMP1*, a member of the family of natural inhibitors of the MMPs, was increased in fibroblasts after treatment with TGF-β (D1+ TGFb). In addition to its inhibitory role against most of the known MMPs, the encoded protein promotes cell proliferation in a wide range of cell types and may also have an anti-apoptotic function. Importantly, *MMP1* and *TIMP1* levels were increased at the mRNA level in induced sputum from asthmatic subjects compared with non-asthmatics^61^. Our data suggest that fibroblasts and myofibroblasts, in the context of the increased TGF-β in the asthmatic airway, may contribute to this change.

Of the genes identified in literature as being relevant to asthma or TGF-β pathways, *ADAMTS1*^21^ and *DSP*^22^ were identified in both real-time PCR and RNASeq analysis as showing significant differences in gene expression between D20+ TGF-β and D20 samples. However, there was no difference in the activity of these genes between D20+ TGF-β and D1+ TGF-β samples. Our data strongly suggest that the increased TGF-β in asthmatic airways can induce changes in these genes, in particular an early increase in expression of *DSP*. Importantly, *ADAMTS1* is decreased in myofibroblasts, though it increases over time in fibroblasts in culture. No change was observed in the total cellular ADAMTS1 protein with or without TGF-β in fibroblasts or myofibroblasts; probably as ADAMTS1 is secreted.

We found three genes whose expression was altered in myofibroblasts and fibroblasts treated with TGF-β that were not previously described to be modulated in airway structural cells or in asthma. The *LDLR* gene was upregulated in myofibroblasts. This family consists of cell surface proteins involved in receptor-mediated endocytosis of specific ligands. Recent work in animal models suggests a role for LDLR in modulation of allergic asthma severity via its recognition of apolipoproteins^62^. Of note, LDLR serves as receptor for a group of rhinoviruses^63^; whether the observed upregulation leads to increased susceptibility of myofibroblasts to these viruses remains to be confirmed. *RHEB* was upregulated in myofibroblasts. RHEB is a key intermediate in the PTEN pathway on which it has an inhibitory effect, and is overexpressed in many cancers where it has been variously associated with increased cell proliferation, hyperplasia and fibrosis^64^. Furthermore, in the current study the increased gene expression of *RHEB* correlated with increased protein expression of RHEB in the presence of TGF-β.

Pathway analysis of the D20+ TGF-β vs D20 samples or D20+ TGF-β vs D1+ TGF-β samples showed that cytoskeleton remodelling (including TGF-β and PKB driven changes) and cell adhesion pathways were altered, as expected. PKB and PTEN signalling pathways were altered in D20+ TGF-β vs D1+ TGF-β samples only. PKB and other intermediates of the mitogen-activated protein kinase (MAPK) signalling pathway are components of the non-canonical pathways induced by TGF-β^65^. TGF-β downregulates PTEN, while activating PKB and extracellular signal-regulated kinase (ERK) pathways in non-small cell lung cancer patients^66^. Importantly, decreased PTEN expression and activity is associated with allergen exposure in a mouse model of bronchial asthma^67^, and increased GRβ suppresses PTEN in cell culture, leading to PKB stimulation^68^. GRβ is increased in transdifferentiated WI-38 cells^14^ and may be responsible for the observed changes in PTEN pathway; however this remains to be confirmed.

Additionally, EGFR signalling and regulation of EIF4F activity were also altered with this treatment as has been described previously^69^. EGFR has an important role in the proliferation of the airway mesenchymal cells. EGFR ligands are released from the damaged epithelium, and act via EGFR on fibroblast cell surface to induce cellular proliferation^28^. Our data showing induction of the EGFR pathway strongly suggests that myofibroblasts proliferation may also be induced by EGF ligand in the airways.

## Conclusion

In conclusion, we have found a significant number of genes that are altered after differentiation of fibroblasts into myofibroblasts by TGF-β treatment, many of which were expected or predicted. We also identified genes that were unexpectedly altered, including *LDLR*, *CSNK2B* and *RHEB*, which suggests different pathways that are activated during the transition between fibroblasts and myofibroblasts, and thus may contribute to the asthma phenotype. We further identified novel intermediates in pathways that were affected after treatment of fibroblasts with TGF-β, including PKB and PTEN signalling pathways. PKB and PTEN pathways are known to be impacted in asthma^70^ and our data suggests that myofibroblasts may contribute to this effect.

The human lung fibroblast WI-38 cell line used in this study represents a promising model to study lung disease and more particularly asthma, in providing better understanding of the contribution of myofibroblast cells to disease phenotype.

## Supplementary information

Supplementary Figures.

## Data Availability

The datasets generated during the current study are available in the Gene Expression Omnibus repository, http://www.ncbi.nlm.nih.gov/geo/query/acc.cgi?acc=GSE110021.
