# Supplementary material for: Transcriptomic changes during TGF-β-mediated differentiation of airway fibroblasts to myofibroblasts
Source: Sci Rep. 2019 Dec 30;9:20377. doi: 10.1038/s41598-019-56955-1 (PMC6937312; doi:10.1038/s41598-019-56955-1)

Transcriptomic changes during TGF-β-mediated differentiation of airway fibroblasts to myofibroblasts.

Erin Joanne Walker, Deborah Heydet, Timothy Veldre[*], Reena Ghildyal[#]

Centre for Research in Therapeutic Solutions, Faculty of Science and Technology, University of Canberra, Bruce, ACT, 2617.

ejwalker08@gmail.com

hdeborah6@hotmail.com

t_veldre@hotmail.com

Reena.Ghildyal@canberra.edu.au

# corresponding author

* present address – Department of Respiratory and Sleep Medicine, The Canberra Hospital, Garran, ACT, 2605.

Supplementary Figure Legends

Figure S1 – Full length blots to accompany Figure 1. WI-38 cells were cultured in presence of TGFβ (2ng/mL, indicated by '+' above the lane) or left untreated (-) for 20 days. Cells were lysed on day 1 and day 20 and analysed for expression of vimentin and αSMA by western blotting as described in the Methods section. Molecular weights for prestained markers (Bioline Hyperpage, lane marked M) are indicated on the left in kDa. Vimentin and αSMA are indicated on the right.

Figure S2 – Immunofluorescence data to complement Figure 1. Immunofluorescence staining for vimentin, αSMA and DAPI in TGFβ treated cells at different times. Day 0 represents cells immediately prior to treatment with TGFβ.

Figure S3 – RNASeq data for GAPDH to accompany Figures 4,5. RNASeq counts per million obtained for *GAPDH* in each sample were log2 transformed and are depicted in the histogram against as average of biological replicates (overall) or as individual biological replicates (e.g. D1-1, D1-2, D1-3). Each biological replicate comprises three technical replicates.

Figure S4 – Correlation between log2 fold change values in RNASeq and in real-time PCR. Log2 fold change values for all genes chosen for real-time PCR validation are shown in the graph. The Pearson coefficient correlation function within XLStat in Excel was used to derive the R^2 value which is shown on the graph.

Figure S1

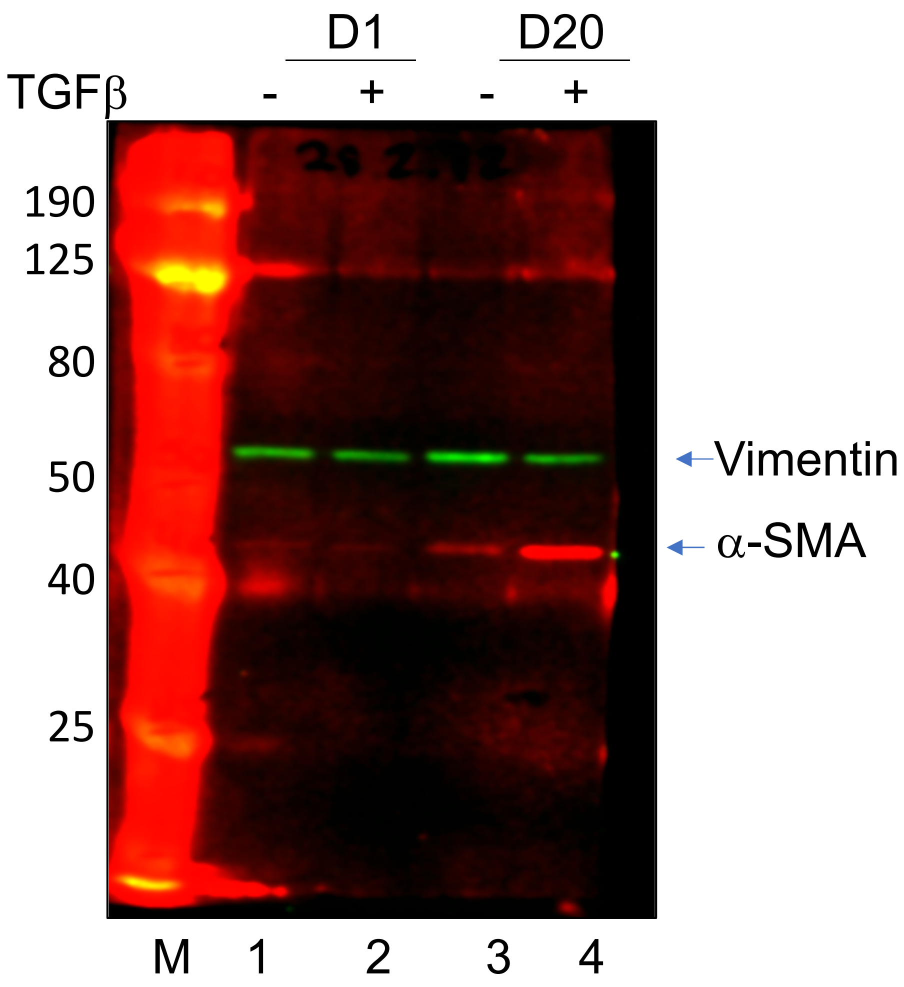

Figure S2

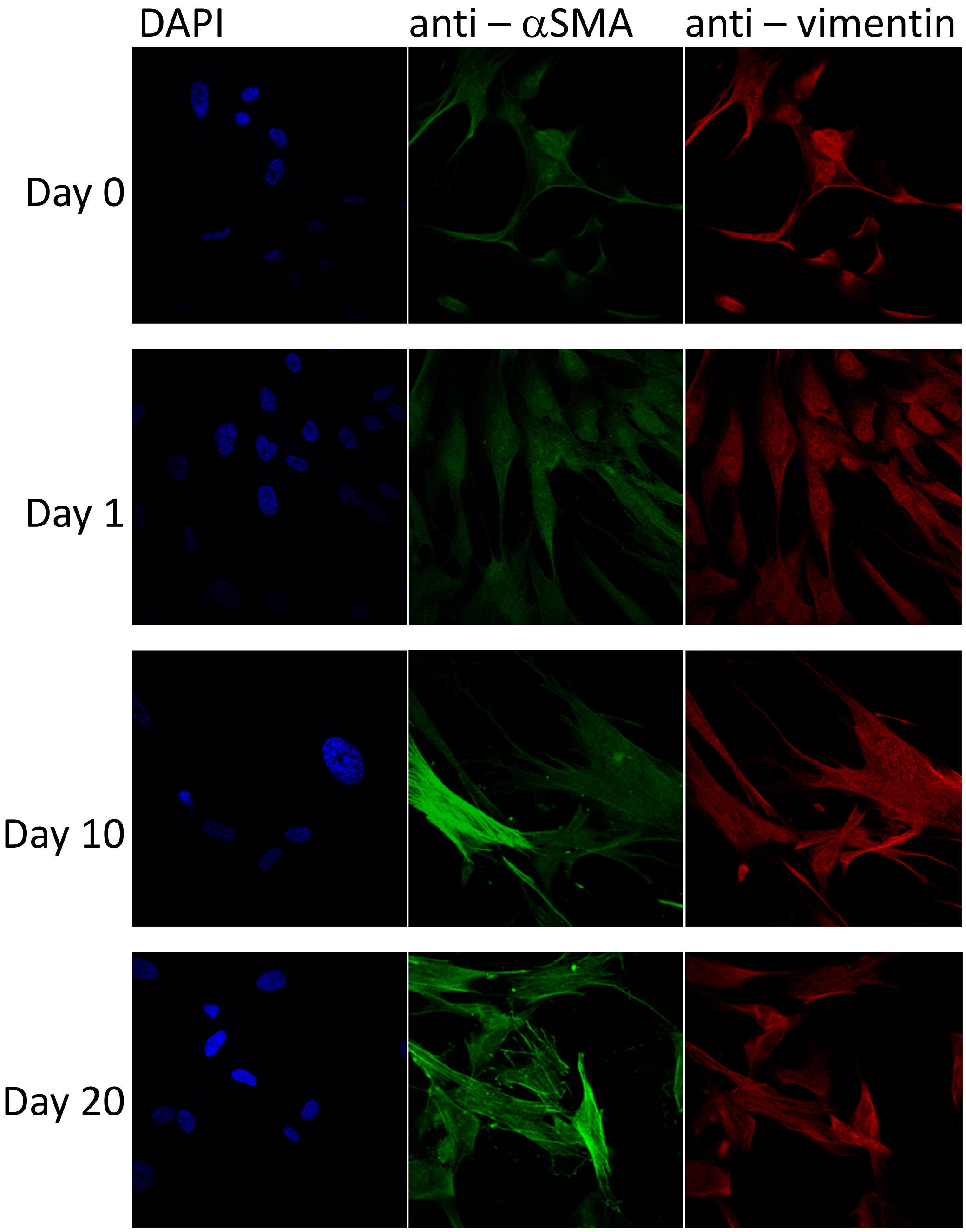

Figure S3

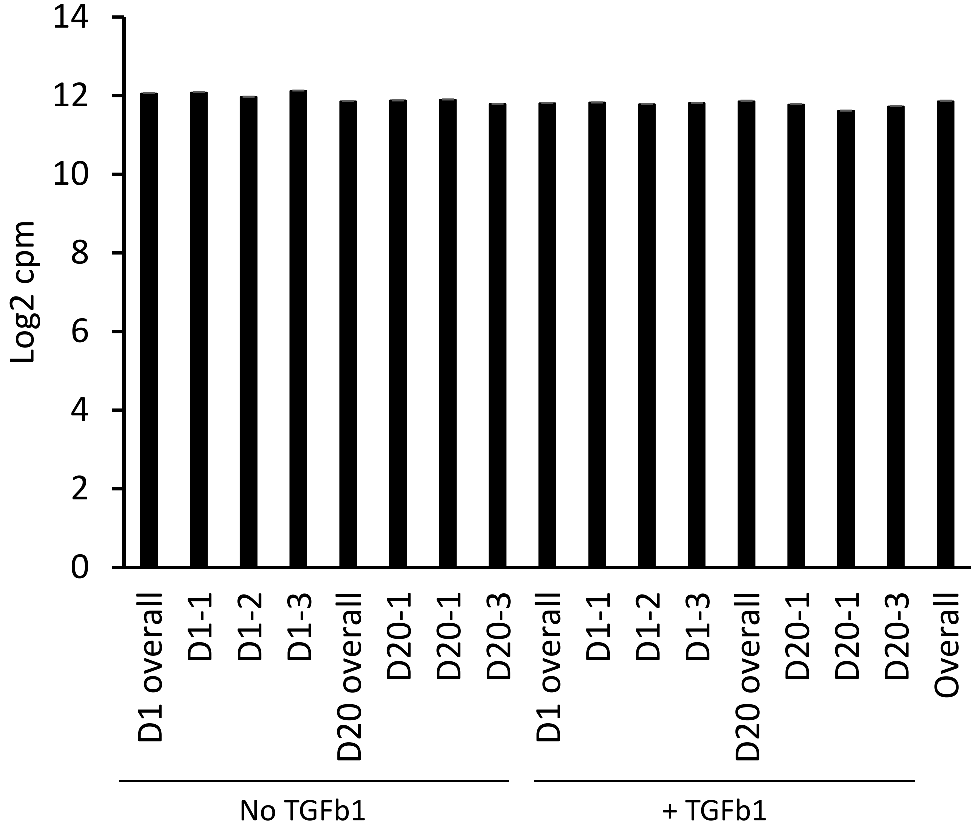

14
12
10
8
6
4
2
0
Log2 cpm
D1 overall
D1-1
D1-2
D1-3
D20 overall
D20-1
D20-1
D20-3
D1 overall
D1-1
D1-2
D1-3
D20 overall
D20-1
D20-1
D20-3
Overall
No TGFb1
+ TGFb1

Figure S4

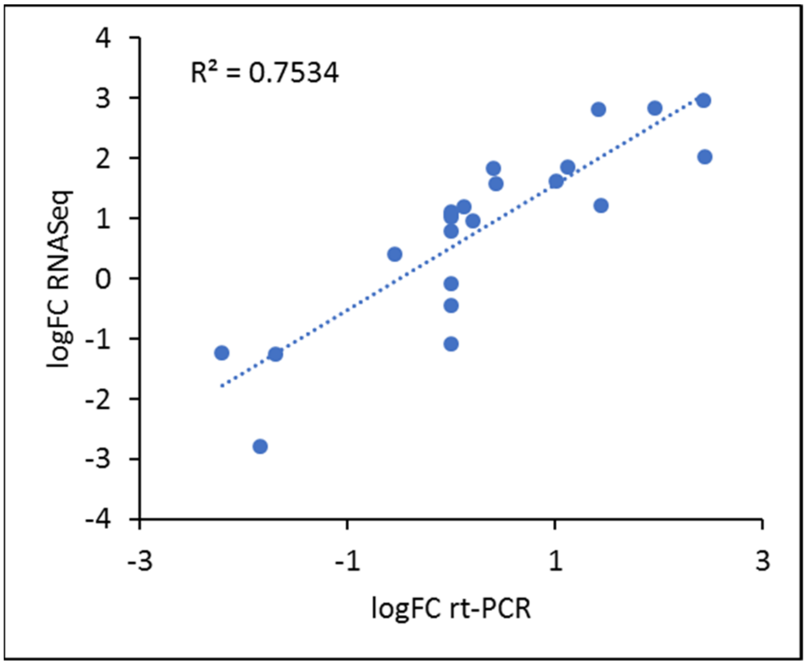

Supplement: Supplementary file 1 — Supplementary Figures. [file 41598_2019_56955_MOESM1_ESM.pdf]
